# Intraoperative Optical Coherence Tomography Analysis of Clear Corneal Incision: Effect of the Lateral Stromal Hydration

**DOI:** 10.1155/2020/8490181

**Published:** 2020-09-08

**Authors:** Jiri Cendelin, Stepan Rusnak, Lenka Hecova

**Affiliations:** ^1^Center of Eye Microsurgery Ofta, Pilsen, Czech Republic; ^2^Department of Ophthalmology, 2nd Faculty of Medicine, Charles University in Prague and Motol University Hospital, Prague, Czech Republic; ^3^Department of Ophthalmology, Faculty of Medicine in Pilsen, Charles University in Prague and University Hospital in Pilsen, Prague, Czech Republic

## Abstract

**Methods:**

The cohort included 65 clear corneal incisions of 49 patients who underwent cataract surgery. Corneal incisions were recorded using a Leica Proveo 8 microscope with an intraoperative OCT EnFocus™ device continuously during the surgery. Corneal incision morphology before and after lateral stromal hydration was analysed.

**Results:**

Good adaptation of the corneal incision before hydration was present in 39 cases (60%), in 16 cases (24.6%), the prominence of posterior lip was present, and, in 10 cases (15.4%), the posterior lip tongue was inverted/retracted into the incision. In 38 cases (58.5%), hydration had no effect on the incision architecture; most often, it was primarily a well-adapted corneal incision (46.2%), less often an incision with posterior lip prominence (10.8%), or tongue inversion into the incision (1.6%) prior to hydration. Hydration worsened the incision architecture in 14 cases (21.5%); most often, it induced/worsened posterior lip prominence (15.4%), less often posterior lip retraction (1.6%), tongue inversion into the incision (1.6%), gap development in the peripheral part of the corneal incision (1.6%), or incomplete opening of the corneal incision (1.6%). In 13 cases (20%), hydration improved the incision architecture, especially in cases with inverted or retracted posterior lip tongue (12.3%), less often in cases with posterior lip prominence (7.7%).

**Conclusion:**

Lateral stromal hydration seldom affects the condition of the corneal incision. Still, it can cause both deterioration and improvement of the corneal incision architecture. Intraoperative OCT provides real-time monitoring of corneal incision morphology during hydration procedure.

## 1. Introduction

Cataract surgery is the most common surgical procedure worldwide, with about 10 million patients undergoing surgery annually [1]. Clear corneal incision (CCI) is currently the most common technique for the construction of an incision in cataract surgery. Stromal hydration of the corneal incision significantly increases incision tightness and reduces postoperative suction of fluid from the eye surface into the anterior chamber [2]. Various techniques of corneal incision closure by hydration have been described [3–6]. Lateral hydration has been performed since the early 1990s [7] and is the most commonly used technique of corneal incision hydration. The technique of lateral stromal hydration consists of gentle irrigation of the balanced salt solution (BSS) into the lateral walls of the incision with visible whitening of the corneal stroma.

For checking the corneal incision tightness after its hydration, the surgeons normally rely on visual examination using an operating microscope and palpation. However, they have no control over whether the stromal hydration has caused distortion of the corneal incision. The visualization of the central orifice of the incision may also be insufficient [8].

Corneal incisions can be easily visualized using anterior segment optical coherence tomography (AS-OCT), used mainly in postoperative period to observe hydration decrease, incision tightness, and presence of complications (e.g., Descemet's membrane detachment, central orifice gaping, and tufted tongue of posterior lip). An intraoperative AS-OCT monitoring of corneal incision during cataract surgery is used rarely; some authors appreciate the possibility of evaluating the morphological features of the corneal incision, such as the length, width, and angle of the incision, respectively, the presence of epithelial disruption, wound gaping, endothelial condition, and Descemet's membrane condition [9–12].

The aim of this prospective study was to assess the effect of lateral stromal hydration on the incision architecture with the help of microscope integrated intraoperative AS-OCT.

## 2. Methods

The group included 65 clear corneal incisions of consecutive cataract surgeries of 49 patients (Table 1). The group consisted of 34 women (69.4%) and 15 men (30.6%); the mean age of the patients was 71.2 years (median 73.0 years). No other ocular diseases were diagnosed in the cohort.

**Figure 1:**
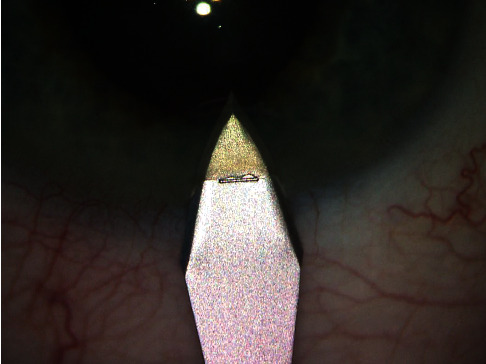
Bevel-up knife with a depth indicator with the reference line 2 mm from the tip.

At the beginning of the procedure, under the topical local anesthesia, 2.6 mm or 2.5 mm perilimbal clear corneal incisions were performed just in front of the capillary line. Corneal incisions were performed with a disposable bevel-up knife (BVI Beaver Xstar Safety Slit Bevel-Up Knife, 2.6 mm or 2.5 mm) with a depth indicator with the reference line 2 mm from the tip. After immersion of the 2 mm knife-tip into the cornea, the knife pointed into the anterior chamber (Figure 1). For the purpose of this study short, long or irregular incisions were excluded.

Patients from the cohort underwent uncomplicated standard cataract surgery (PROVEO Leica Microsystems microscope, Stellaris® ELITE™ Bausch & Lomb microsurgical system); all operations were performed by one surgeon. Preoperatively, all patients signed standard informed consent for cataract surgery. The study was performed in accordance with the Declaration of Helsinki for Human Research.

Corneal incisions were closed by the method of lateral stromal hydration with a curved cannula with BSS. When checking the wound tightness, the wound area was dried and the intraocular pressure was temporarily increased (by irrigating of BSS into the anterior chamber); in cases of ambiguity, a Seidel test was performed with a sterile fluorescein strip. Corneal incisions were recorded using a Leica Proveo 8 microscope with an intraoperative OCT EnFocus™ device (Bioptigen Inc., Leica Microsystems Company, NC) continuously during the surgery. For the purpose of this study, an Ultra-Deep OCT unit was used with a display of up to 11 mm in depth and a resolution degree of ≤9 micrometres. Corneal incisions were continuously monitored with AS-OCT during the surgery. Images covering the entire area of the incision just before and after hydration were selected from the records and evaluated. Longitudinal scans were evaluated before and after hydration at the site of the worst adaptation of the incision.

**Figure 2:**
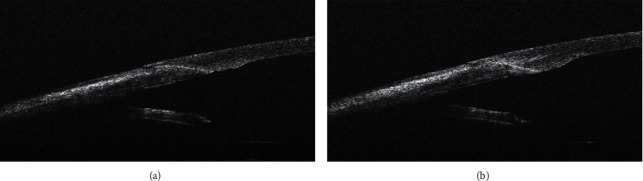
Well-adapted corneal incision before hydration (a); lateral stromal hydration had no effect on corneal incision architecture (b).

**Figure 3:**
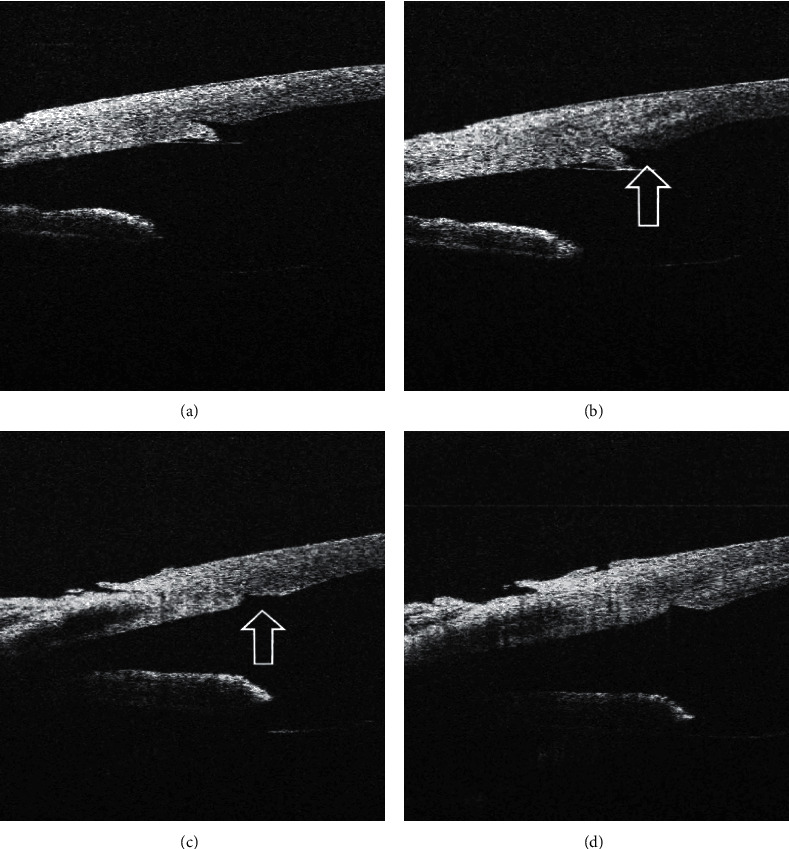
Prominence of posterior lip of the corneal incision before (a) and after (b) hydration, tongue inversion into the incision before (c) and after (d) hydration. In both cases, lateral stromal hydration had no significant effect on corneal incision architecture. The arrow shows the exposed central part of the incision ceiling; this may explain the gap of the incision orifice visible with a operating microscope (see Figure 3(b) corresponds with 5(a), respectively, and 3(c)with 5(b)).

**Figure 4:**
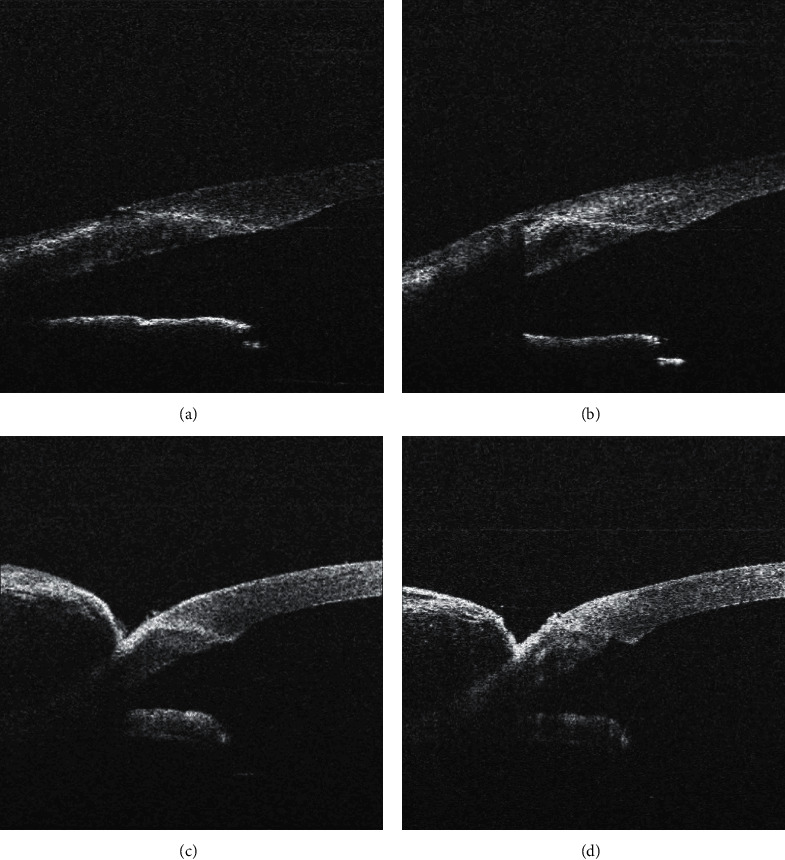
Worsening of corneal incision morphology after its hydration. Well-adapted corneal incision before hydration (a), anterior lip prominence centrally from the inner orifice developed after lateral stromal hydration (b). Tongue inversion into the incision before (c) and after (d) lateral stromal hydration.

As well-adapted incision, the cases with the attached ceiling and floor in the entire extent of the incision were considered (Figure 2(a)). The cases with eversion or detachment of the central part of the lower lobe towards the anterior chamber were described as an incision with prominence of the posterior lip (Figure 3(a)). Incisions with tongue inversion (Figure 3(c)) were cases with inversion of the central part of the posterior lip towards the wound. Finally, tongue retraction (Figure 4) was described in cases where there was an apparent shortening of the lower lobe with exposure of the central part of the incision ceiling towards the anterior chamber.

The study dealt only with the possibility of the actual influence of lateral stromal hydration on corneal incision morphology. In cases of the incision deformation or leakage after hydration, the incision shape was adjusted or additional hydration was performed.

## 3. Results

The results are summarized in Table 2.

### 3.1. Condition of Corneal Incision before Hydration

Good adaptation of the corneal incision (i.e., well-adapted posterior lip without signs of retraction or prominence, good tightness of the incision) was observed in 39 cases (60%), in 16 cases (24.6%), different prominence of the posterior lip was observed, and, in 10 cases (15.4%), tongue inversion into the incision or tongue retraction was observed.

### 3.2. Influence of Lateral Hydration on Architecture of Corneal Incision

**Figure 5:**
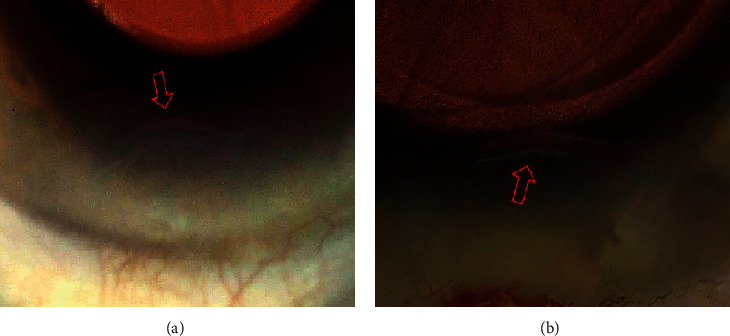
Photos of corneal incisions: the gap of the incision orifice (exposed central part of the incision ceiling) seen in photo from standard intraoperative video with different OCT findings (see Figure 3).

In 38 cases (58.5%), lateral stromal hydration had no effect on the corneal incision architecture; 30 of them (46.2%) were primarily well-adapted corneal incisions (Figure 2); in 7 cases (10.8%), prominence of posterior (internal) lip and, in one case, (1.6%) tongue inversion into the incision prior to hydration had been presented (Figures 3 and 5).

**Figure 6:**
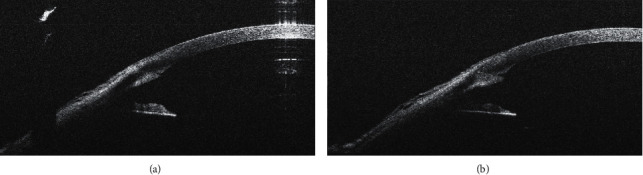
Worsening of corneal incision morphology after its hydration. Posterior lip prominence presented before hydration (a) worsened after lateral stromal hydration (b).

**Figure 7:**
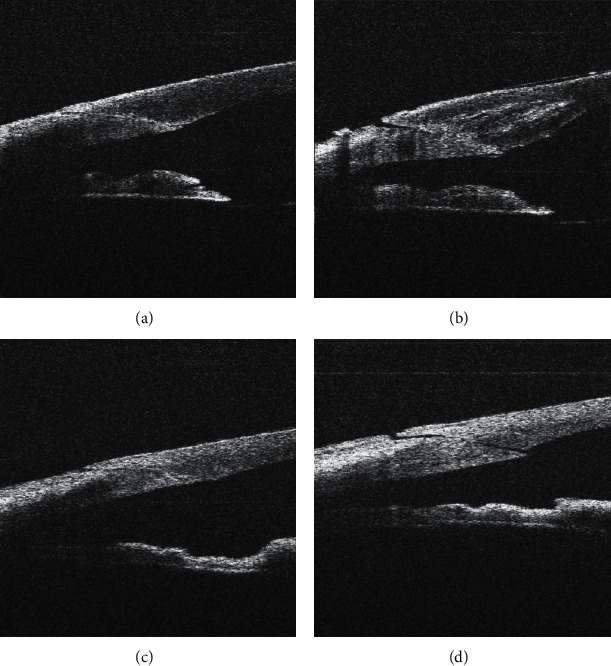
Worsening of corneal incision morphology after its hydration. Well-adapted corneal incision before hydration (a); gap development in the corneal incision periphery after lateral stromal hydration (b). Well-adapted corneal incision before hydration (c); corneal incision almost opened longitudinally after lateral stromal hydration (d).

In 14 cases (21.5%), lateral stromal hydration worsened the condition of the corneal incision. In 10 cases (15.4%), it was the development of posterior lip prominence (of which 4 were slightly present before the incision hydration but lateral hydration worsened the condition of the posterior lip) (Figure 6), in one case (1.6%), hydration led to anterior lip prominence centrally from the inner orifice, in one case (1.6%), hydration worsened the tongue inversion in the incision (Figure 4), in one case (1.6%), a gap developed in the periphery of the corneal incision, and, in one case (1.6%), more than two-thirds of the incision opened longitudinally (Figure 7).

**Figure 8:**
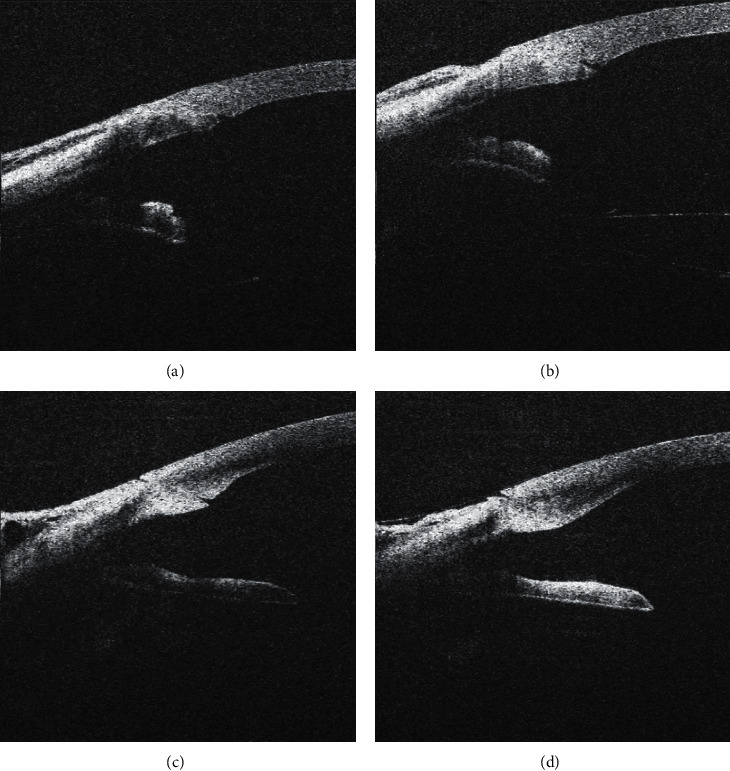
Improvement of corneal incision morphology after its hydration. Inverted tongue of the corneal incision before hydration (a) and after lateral stromal hydration inverted tongue strengthened up and slight prominence of posterior lip appeared (b). Prominence of the posterior lip (c); decrease after lateral stromal hydration (d).

In 13 cases (20%), lateral stromal hydration improved the corneal incision architecture. In 8 cases (12.3%), after hydration, the inverted or retracted tongue of the corneal incision was strengthened and adapted or was slightly protruding; in 5 cases (7.7%), the prominence of the posterior lip decreased after hydration (Figure 8).

Wound leakage after hydration occurred in two cases; in one case of tongue inversion (Figure 4(d)), the leakage stopped after the posterior lip was adjusted manually; in the second case (Figure 7(d)), the leakage was eliminated by increasing of the intraocular pressure for 30 seconds).

## 4. Discussion

Sutureless clear corneal incision (CCI) is the most common method for incision wound constructing in cataract surgery [1]. However, this type of incision is associated with an increased risk of postoperative endophthalmitis development [13–15]. According to Taban et al. [14], the risk of postoperative endophthalmitis in corneal incision is 0.189%, compared to 0.074% in scleral incision, respectively, 0.062% in limbal incision. Microorganisms have two main ways of how to get into the eye and cause infection, either during the surgery itself or in the early postoperative period before epithelialization of the sutureless corneal incision. Stromal hydration of the corneal incision reduces the risk of postoperative endophthalmitis development, increases incision tightness, and reduces intraocular fluid leakage through the incision to the eye surface, while reducing postoperative suction of fluid from the eye surface into the anterior chamber. Vasavada et al. [2] applied 0.0125% trypan blue to the eye surface with and without hydration of the corneal incision and detected a statistically significantly lower (*p* < 0.001) concentration of trypan blue in the anterior chamber of the hydrated incision group.

There are two main techniques for corneal incision closure by hydration. Conventional lateral stromal hydration consists of gentle irrigation of the balanced salt solution (BSS) into the lateral walls of the incision, followed with a visible whitening of the corneal stroma [7]. Other possibility is the hydration into the supraincisional pocket (the so-called Wong incision), i.e., an additional incision performed in the anterior lip in front of the original corneal incision with a corneal knife to a depth of approximately 160 *μ*m [5]. According to Mifflin et al. [3], hydration in the supraincisional pocket is significantly better than conventional lateral stromal hydration in preventing wound leakage due to direct pressure on the posterior lip of the corneal incision. However, this method may carry an additional risk of epithelial damage development and increased astigmatism [3].

The tightness of a corneal incision after its hydration is normally assessed visually (visible fluid leakage from the wound, corneal stroma whitening after hydration, presence of flaccid posterior lip, etc.) and with palpation eventually with a modified Seidel test [4]. The visualization of the central orifice of the incision can be also insufficient [8]. Calladine and Packard [16] and Behrens et al. [17], respectively, draw attention to the risk of corneal incision leakage after hydration has ceased. The persistence of hydration of the corneal incision is a subject of further discussion [9–11].

Corneal incisions can be easily visualized by AS-OCT; it is especially used in the postoperative period to monitor the loss of hydration [9, 11], tightness of the incision, and the presence of complications. Calladine and Tanner [10] investigated the effect of stromal hydration on corneal incision architecture using an AS–CT; if measured 1 hour after stromal hydration, it significantly increases the length of the corneal incision and it is associated with an increased incidence of local Descemet's membrane detachment compared to incision without hydration. Bang et al. [11] performed corneal incision analysis using OCT 2 hours, 1 day, 1 week, 1 month, and 3 months after surgery and compared the effect of lateral stromal hydration on the incision architecture. In addition to corneal thickness, incision length (2.2 mm and 2.8 mm, resp.), and angle, he observed the presence of epithelial or endothelial wound gaping and local Descemet's membrane detachment. According to the results of this study, smaller corneal incisions (2.2 mm) are more vulnerable to external influences (e.g., stromal hydration) and less stable than larger incisions.

Several authors are interested in the use of intraoperative anterior segmental OCT in anterior segment surgery. In 2018, Ehlers et al. [18] published the 3-year results of the DISCOVER study, which focuses on the feasibility and benefits of microscope integrated OCT during various ophthalmological surgeries. Intraoperative OCT appears to be a useful aid for anterior segment surgery, especially in positioning of DSAK, DMEK, and intracorneal inlays. In 43.4% of anterior segment operations, the surgeons concluded that the current information that they had continually acquired during surgery had an impact on their surgical decisions and altered the surgical procedure. Titial et al. [19] studied morphology of the inner orifice of the corneal incision using an intraoperative AS-OCT and conducted an irregular incision to be a predisposition for local Descemet's membrane detachment. The highest incidence of local Descemet's membrane detachment was observed during final stromal hydration of the incision. Almutlak et al. [20] presented the use of microscope integrated AS-OCT; the authors were able to follow the corneal wound architecture, its development during the procedure, and the condition of the Descemet's membrane. Das et al. [21] used intraoperative AS-OCT in both microincision and femtosecond laser assisted cataract surgery. Using AS-OCT, the surgeon could actually evaluate the morphological features of the corneal incision, such as the length, width, and angle of the incision, respectively, the presence of epithelial disruption, wound gaping, endothelial condition, and Descemet's membrane condition. The authors also marginally mention the possibility of the intraoperative monitoring (using AS-OCT) of stromal hydration efficacy and adequacy. However, they do not deal with the effect of hydration on the corneal wound morphology in detail.

In our cohort, corneal incisions were continuously monitored using intraoperative AS-OCT. The aim of our work was to evaluate the morphology of the incision before and after hydration. According to the results of our study, lateral hydration least affects primarily well-adapted corneal incisions. Even in the cases of primarily well-adapted incisions, the effect of the lateral hydration may not be ideal and hydration may lead to posterior lip prominence, less often to anterior lip prominence centrally from the inner orifice, tongue inversion into the incision, gap development in the corneal incision periphery, or even to its incomplete opening. In cases of tongue inversion into the corneal wound, lateral stromal hydration may cause worsening of the inversion. In cases of prominence of the posterior lip, lateral hydration does not alter, worsen, or normalize the wound morphology approximately equally.

The primary condition of the incision before the hydration seems to be the most important factor. Distortion of the incision can be caused by different straining, deformations, and self-hydration during the surgery. With an unarmed microscope without AS-OCT, the surgeon may have limited the possibility of assessing the actual condition of the incision.

Lateral hydration may improve or worsen the corneal incision morphology at the end of the surgery, which may be the cause of clinically apparent failure, i.e., persistence or new leakage of the corneal incision after its hydration.

Our study demonstrates the value of microscope integrated intraoperative OCT in perioperative evaluation of corneal incisions. The possibility of detecting tongue inversion into the incision, which may not be noticeable due to the clouding after corneal incision hydration and may lead to incision incompetence, seems to be the most useful [8]. Intraoperative OCT provides an excellent opportunity to monitor the corneal incision morphology in real time and allows the surgical procedure in the hydration phase to be modified dynamically and purposefully to achieve the best tightness and architecture of the corneal wound.

## Figures and Tables

**Table 1:** Patients group characteristics.

Group of patients	49 patients	65 corneal incisions
Gender	Women	Men
	34 (69.4%)	15 (30.6%)

Age	Mean	Median
	71.2 years	73.0 years

**Table 2:** Condition of corneal incision before and after hydration.

	Before hydration		After hydration					
			Well-adapted	Posterior lip prominence	Tongue inversion/retraction	Anterior lip prominence	Gap development	Incision opening
Well-adapted	39 (60%)	>	30 (46.2%)	6 cases (9.6%)	1 (1.6%)	0	1 (1.6%)	1 (1.6%)

Posterior lip prominence	16 (24.6%)	>	5 (8%)	10 (16%)	0	1 (1.6%)	0	0

Tongue inversion/retraction	10 (15.4%)	>	8 (12.8%)	0	2 (3, 2%)	0	0	0

## Data Availability

The data used to support the findings of this study are included within the article.
